# An Ensemble Method to Distinguish Bacteriophage Virion from Non-Virion Proteins Based on Protein Sequence Characteristics

**DOI:** 10.3390/ijms160921734

**Published:** 2015-09-09

**Authors:** Lina Zhang, Chengjin Zhang, Rui Gao, Runtao Yang

**Affiliations:** 1 School of Control Science and Engineering, Shandong University, Jinan 250061, China; E-Mails: zlnabc2010@163.com (L.Z.); gaorui@sdu.edu.cn (R.G.); runtao-sd@163.com (R.Y.); 2 School of Mechanical, Electrical and Information Engineering, Shandong University, Weihai 264209, China

**Keywords:** bacteriophage virion proteins, ensemble method, hybrid features, relief

## Abstract

Bacteriophage virion proteins and non-virion proteins have distinct functions in biological processes, such as specificity determination for host bacteria, bacteriophage replication and transcription. Accurate identification of bacteriophage virion proteins from bacteriophage protein sequences is significant to understand the complex virulence mechanism in host bacteria and the influence of bacteriophages on the development of antibacterial drugs. In this study, an ensemble method for bacteriophage virion protein prediction from bacteriophage protein sequences is put forward with hybrid feature spaces incorporating CTD (composition, transition and distribution), bi-profile Bayes, PseAAC (pseudo-amino acid composition) and PSSM (position-specific scoring matrix). When performing on the training dataset 10-fold cross-validation, the presented method achieves a satisfactory prediction result with a sensitivity of 0.870, a specificity of 0.830, an accuracy of 0.850 and Matthew’s correlation coefficient (MCC) of 0.701, respectively. To evaluate the prediction performance objectively, an independent testing dataset is used to evaluate the proposed method. Encouragingly, our proposed method performs better than previous studies with a sensitivity of 0.853, a specificity of 0.815, an accuracy of 0.831 and MCC of 0.662 on the independent testing dataset. These results suggest that the proposed method can be a potential candidate for bacteriophage virion protein prediction, which may provide a useful tool to find novel antibacterial drugs and to understand the relationship between bacteriophage and host bacteria. For the convenience of the vast majority of experimental scientists, a user-friendly and publicly-accessible web-server for the proposed ensemble method is established.

## 1. Introduction

A bacteriophage, living within a host bacterium, is a virus that consists of DNA or RNA, structural proteins and a few other packaged proteins [1]. It replicates itself by making use of the host biosynthetic machinery, infects host bacterium and eventually destroys or hijacks the bacterial cell [2]. Bacteriophages play some positive, as well as negative roles in biology, ecology and health. In the positive field, bacteriophages are extensively applied to bacterial identification, bacterial pathogenesis, organismal ecology and evolutionary biology [3]. In particular, clinical phage therapy aiming at different bacterial diseases may provide a novel perspective to deal with the growing threat of antibiotic resistance in pathogenic bacteria [4]. On the other hand, the pollution of bacteriophages could cause a great loss to the fermentation industry.

Bacteriophage proteins are of vital importance to determine the functions and mechanisms of bacteriophages. Bacteriophage proteins include structural (virion) proteins and non-structural (non-virion) proteins. Bacteriophage virion proteins, coded by the bacteriophage genome, are components of the mature assembled bacteriophage particles. They may include capsid proteins, envelope proteins and virus particle enzymes [5]. These virion proteins determine the specificity for recognizing host bacteria and perform great functions in bacteriophage virus recombination, receptor recognition, bacteria attachment and penetration [6]. Bacteriophage non-virion proteins, also encoded by the bacteriophage genome and produced in infected cells, are not packaged in the mature bacteriophage particles [5]. These non-virion proteins, mostly enzymes and regulatory proteins, play important roles in bacteriophage replication, transcription and polyprotein processing [7].

Due to the major contributions of virion proteins in deciding the functions and applications of bacteriophages, identifying bacteriophage virion proteins is crucial to gain insight into the relationship between bacteriophage and host bacteria, as well as the influence of bacteriophages on the development of new pathogens and antibacterial drugs. The prediction of virion components of the bacteriophages based on the homology of genomic data leads to bad results [8]. With the avalanche of genome sequences generated in the post-genomic age, more than 70% of bacteriophage sequences in the viral reference sequence database that encode proteins do not have annotated functions [1]. Therefore, it would be urgent to develop computational methods for rapidly and effectively identifying bacteriophage virion proteins from bacteriophage sequences.

In recent years, several computational methods have been reported for bacteriophage gene function prediction and virion protein prediction. Li *et al*. [9] developed the novel support vector machine-based system called SynFPS to perform gene function prediction, which utilized gene-to-gene distances based on the K-means clustering technique to identify closely-related genomes. Feng *et al*. [10] developed a naive Bayes-based predictor to identify bacteriophage virion proteins, using primary amino acid and dipeptide composition as the encoding scheme. Later, Ding *et al*. [11] utilized support vector machine to establish the prediction model for bacteriophage virion protein prediction. In this model, ANOVA (analysis of variance) was applied to select the high discriminative features derived from g-gap dipeptide compositions. The aforementioned methods have their own merits and achieve acceptable results, but the following shortcomings still need to be taken into consideration. (1) Previous studies relied on composition-based features, which were extracted merely based on the alphabetic sequence of the amino acids, but failed to capture some useful features based on physicochemical properties, evolutionary information, *etc*. In general, a single feature cannot preserve enough discriminative information for protein attribute predictions, and multiple features can complement each other to enhance the performance and robustness of a predictor [12,13]. In view of the above-mentioned fact, hybrid features have been increasingly used in recent studies instead of single features for constructing classifiers [14,15]; (2) The existing methods were all based on individual classifiers, which could have their own inherent defects, leading to unsatisfactory predictions [16]. It is generally accepted that the ensemble predictor, which integrates multiple basic classifiers of diverse learning policies (or diversely trained), can achieve better prediction performance than its component classifiers for protein attribute predictions [17]. Thus, the ensemble predictor has been considered as the future direction for protein classification problems [18].

**Figure 1:**
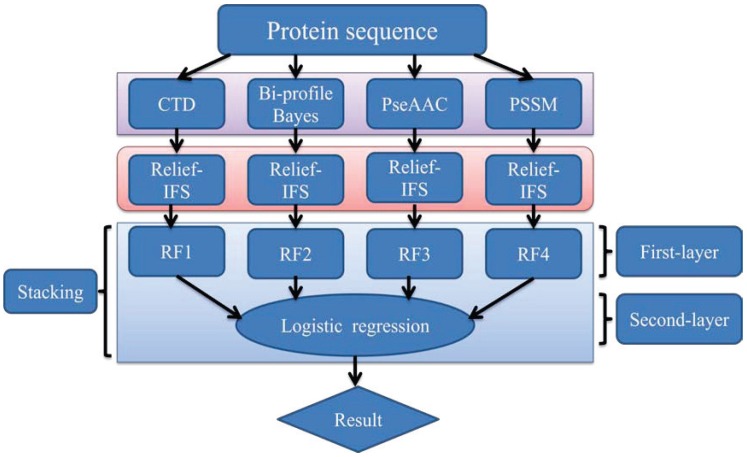
The computational framework of the proposed method. CTD: composition, transition and distribution; PseAAC: pseudo-amino acid composition; PSSM: position-specific scoring matrix; IFS: incremental feature selection; RF: random forest.

In order to address the above-mentioned limitations and to improve the performance for bacteriophage virion protein prediction, this study puts forward a stacking-based ensemble method with hybrid features incorporating CTD (composition, transition and distribution), bi-profile Bayes, PseAAC (pseudo-amino acid composition) and PSSM (position-specific scoring matrix). The proposed method is implemented in the following steps. (1) The benchmark dataset is obtained from Universal Protein Resource (UniProt) and divided into a training dataset and an independent testing dataset; (2) The protein sequences are converted into four different numerical feature vectors based on CTD, bi-profile Bayes, PseAAC and PSSM, respectively; (3) The relief algorithm combined with incremental feature selection (IFS) method is adopted to obtain optimal feature subsets for individual feature spaces; (4) The ensemble method is then developed by stacking the predictions of individual classifiers trained by the above-mentioned distinct feature spaces. A summary of the computational framework of our method is illustrated in Figure 1. To evaluate the performance of our ensemble predictor objectively, the predictive capability of the present model is tested on the independent testing dataset and compared with that of [10,11].

## 2. Results and Discussions

### 2.1. Performance Analysis of RF Models Using Individual Feature Spaces

RF models are constructed based on the training dataset by 10-fold cross-validation to obtain the corresponding optimal parameters of individual feature spaces, including CTD, bi-profile Bayes, PseAAC and PSSM.

**Figure 2:**
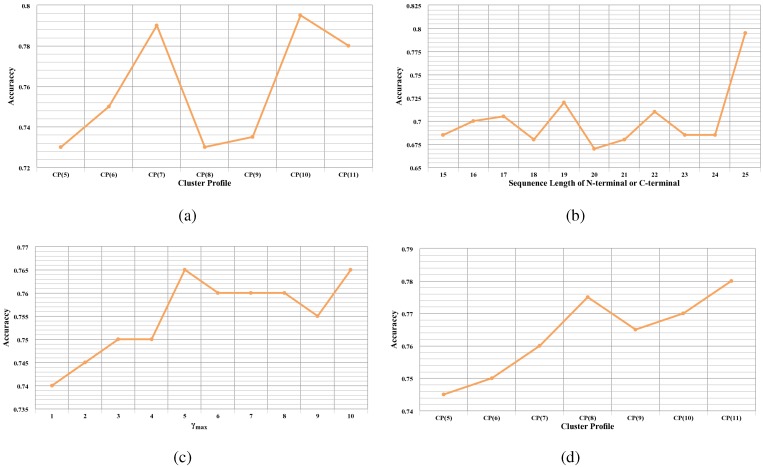
Prediction accuracy of individual feature spaces with different parameters. (**a**) Prediction accuracy of CTD with different cluster profiles; (**b**) prediction accuracy of bi-profile Bayes with different lengths of the N-terminus and C-terminus; (**c**) prediction accuracy of PseAAC with different tier correlation factors γ; (**d**) prediction accuracy of PSSM with different cluster profiles.

Using CTD features, the accuracies of the RF model using CTD features with different cluster profiles are demonstrated in Figure 2a. The best accuracy of 0.795 is achieved when the predictions are based on CP(10), illustrating the good capability for predicting bacteriophage virion proteins. With CP(10), the MCC value of 0.590 shows a reasonable balance between the sensitivity of 0.78 and the specificity of 0.81.

Figure 2b presents the accuracies of the RF model using bi-profile Bayes with different lengths of the N-terminus and C-terminus variably from 15 to 25. The highest accuracy of 0.795 is achieved with 25 amino acids on the N-terminus and C-terminus, as shown in Figure 2b. Meanwhile, the MCC of 0.595 and the AUC of 0.835 indicate reasonably good performance of the RF model using these features.

The prediction performance of the RF model using PseAAC with different γ varying from 1 to 10 is illustrated in Figure 2c. As can be seen from Figure 2c, the RF model obtains the best accuracy of 0.765 at γ=5. The sensitivity of 0.79 and the specificity of 0.74 represent that true positives and true negatives predicted by the feature space are promising and basically balanced. Other performance measures also reveal the discrimination power of PseAAC for the training dataset.

Figure 2d demonstrates the performance of the RF model using PSSM features based on different cluster profiles. The highest accuracy of 0.78 is achieved with CP(11) displayed in Figure 2d. Meanwhile, the MCC of 0.564 and the AUC of 0.805 manifest the satisfactory results based on these features.

**Figure 3:**
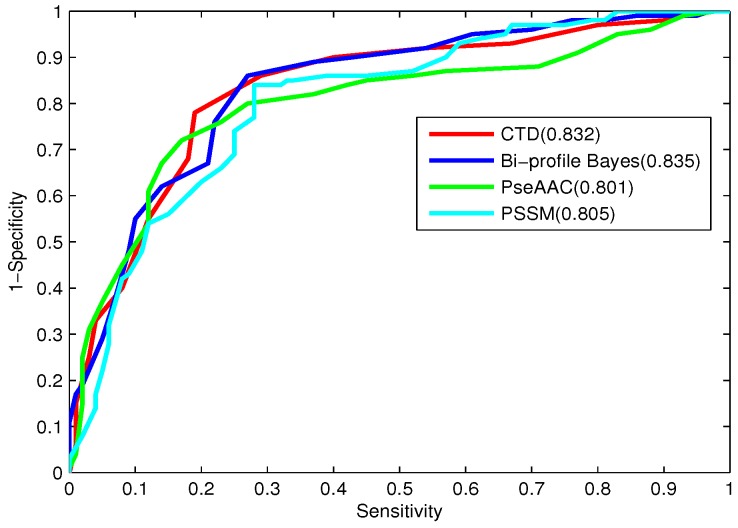
ROC curves of the best-performing individual feature spaces.

In order to compare the predictive power of each feature model, the 10-fold cross-validation results obtained by the RF models using individual feature spaces on the training dataset are listed in Table 1. The ROC curves of the four feature encoding models are shown in Figure 3. As shown in Table 1 and Figure 3, all four feature spaces possess reasonable discrimination power for bacteriophage virion proteins. Bi-profile Bayes achieves the best performance among the four feature spaces with an accuracy of 0.795, an MCC of 0.595 and an AUC of 0.835, due to it taking into account the statistical characterizations of peptide sequences on both the N-terminus and C-terminus. CTD contains the composition, transition and distribution information based on physicochemical properties along the protein sequence, which results in satisfactory prediction results. PSSM, considering not only the physicochemical properties, but also preserving the evolution information of the protein sequence, yields acceptable prediction performance. What is more, the discrimination power of PseAAC is less compared to that of the other three feature spaces, due to the fact that the sequence order information based on physicochemical properties along the sequence may not have enough information for identifying bacteriophage virion proteins.

**Table 1:** Performance of RF models trained with the best-performing individual feature spaces on the training dataset by 10-fold cross-validation. MCC, Matthew’s correlation coefficient.

Feature Space	Sensitivity	Specificity	Accuracy	MCC	AUC
CTD	0.78	0.81	0.795	0.590	0.832
Bi-profile Bayes	0.86	0.73	0.795	0.595	0.835
PseAAC	0.79	0.74	0.765	0.531	0.801
PSSM	0.84	0.72	0.78	0.564	0.805

### 2.2. Performance Analysis of RF Models Using Individual Feature Spaces with Feature Selection

**Figure 4:**
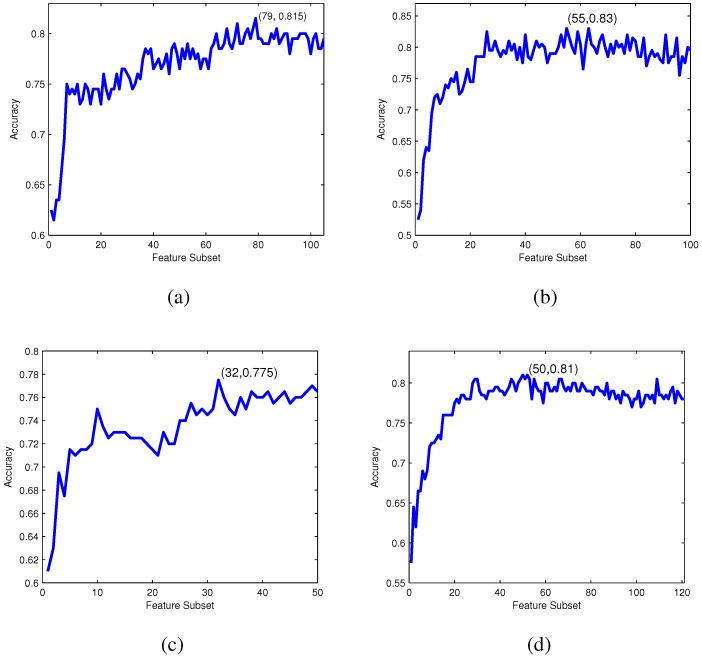
IFS curves for CTD, bi-profile Bayes, PseAAC and PSSM. (**a**) The IFS curve: the values of accuracy against the CTD feature subsets; (**b**) the IFS curve: the values of accuracy against the bi-profile Bayes feature subsets; (**c**) the IFS curve: the values of accuracy against the PseAAC feature subsets; (**d**) the IFS curve: the values of accuracy against the PSSM feature subsets.

The ranked feature lists of four feature spaces are obtained according to their relevance to the classes based on the Relief method. Within the lists, a feature with a smaller index represents a more important one for bacteriophage virion protein prediction. The four feature lists are utilized to select the optimal feature subsets in the following IFS procedure, respectively. Based on the four ranked feature lists, adding the ranked features one by one, individual predictors for different feature subsets are constructed using the RF model and evaluated by 10-fold cross-validation, respectively. Then, four IFS curves for CTD, bi-profile Bayes, PseAAC and PSSM are plotted in Figure 4, which shows the relationships of feature indices against the accuracy obtained from the corresponding predictors. Figure 4a represents the IFS curve of accuracy against CTD. The curve reaches its peak at 0.815 when the top 79 features are used. Thus, these 79 features are regarded as the optimal feature subset for CTD. Similar results can also be found in Figure 4b–d. For bi-profile Bayes, PseAAC and PSSM, the maximum accuracy values are 0.83, 0.775 and 0.81 with the top 55, 32 and 50 features, respectively. These features are chosen as the corresponding optimal feature subsets.

Table 2 summarizes the prediction performance of four individual feature encoding models with feature selection. From Table 2, the four RF models with feature selection all perform with promising prediction results, and it can be observed that the bi-profile Bayes model with optimal features yields the highest performance with a sensitivity of 0.83, a specificity of 0.83, an accuracy of 0.83 and an MCC of 0.66.

**Table 2:** Performance of the RF models using the individual feature spaces with feature selection on the training dataset by 10-fold cross-validation.

Feature Space	No. of Features	Sensitivity	Specificity	Accuracy	MCC	AUC
CTD	79	0.81	0.82	0.815	0.63	0.845
Bi-profile Bayes	55	0.83	0.83	0.83	0.66	0.888
PseAAC	32	0.77	0.78	0.775	0.55	0.823
PSSM	50	0.84	0.78	0.81	0.62	0.822

In Table 1 and Table 2, the prediction results of the CTD model using the optimal features are all better than those of the model using all features. As for the bi-profile Bayes model, although the sensitivity with the optimal 55 features is slightly lower than that with all 100 features, other prediction results are all better than those without feature selection. The PseAAC model has similar comparison results as the bi-profile Bayes model. With the optimal features, the PSSM model achieves the same sensitivity as that of the model without feature selection, but higher specificity, accuracy and MCC.

**Figure 5:**
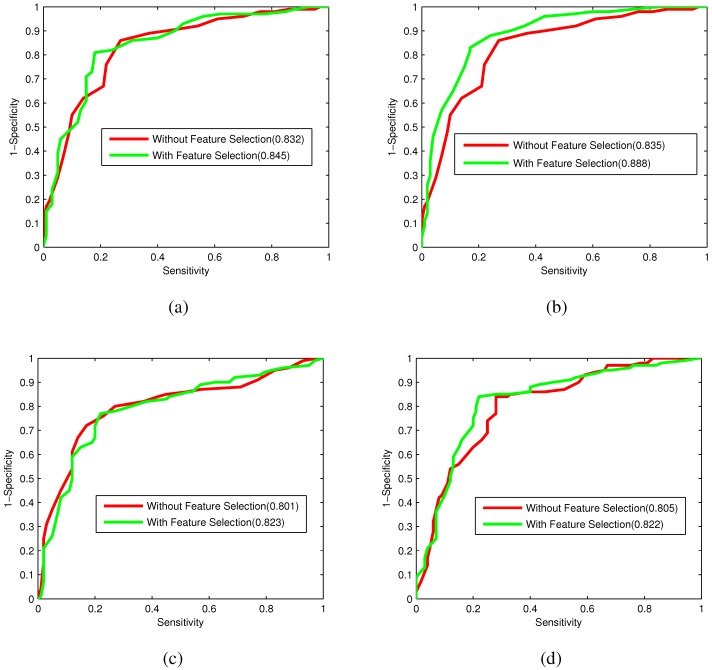
ROC curves for CTD, bi-profile Bayes, PseAAC and PSSM with and without feature selection. (**a**) ROC curves of the CTD model with and without feature selection; (**b**) ROC curves of the bi-profile Bayes model with and without feature selection; (**c**) ROC curves of the PseAAC model with and without feature selection; (**d**) ROC curves of the PSSM model with and without feature selection.

In order to compare the performance of RF models for individual feature spaces with or without feature selection more intuitively, the ROC curves for each of the four feature encoding models with or without feature selection are depicted in Figure 5. From Figure 5, the four feature models for CTD, bi-profile Bayes, PseAAC and PSSM with feature selection all perform better than those without features selection. Therefore, the Relief combined with IFS is effective to eliminate irrelevant and redundant features to improve the predictive performance.

### 2.3. Performance Analysis of the Ensemble Learning Methods

#### 2.3.1. Comparison of Stacking-Based Ensemble Learning and Majority Voting-Based Strategy

As mentioned above, four RF models are trained using the individual feature spaces with feature selection presented in Table 2. Based on the results of individual RF modules, majority voting and stacking are utilized to incorporate them into a consensus classifier to give the final predictions.

The prediction performance of the ensemble learning methods with stacking and majority voting scheme is given in Table 3. As shown in Table 3, though the sensitivity obtained by stacking is 0.01 lower than that yielded by majority voting, other measures are much better than those achieved by the majority voting scheme. It is worth mentioning that the specificity and accuracy obtained by stacking are 0.13 and 0.06 higher than those yielded using the majority voting scheme, which may be attributed to the discrimination power of stacking and the idea of divide and conquer. Compared to the performance in Table 2, the performance achieved by stacking outperforms that obtained by individual feature spaces, while the prediction results yielded by the majority voting-based ensemble method are not better or even worse than those achieved by individual feature spaces. These comparison results reveal that the stacking-based ensemble method is more effective than the majority voting scheme in bacteriophage virion protein prediction. Therefore, this study employs the stacking-based ensemble method to construct the final prediction model.

**Table 3:** Performance of the stacking-based ensemble learning and majority voting-based strategy.

Ensemble Method	Sensitivity	Specificity	Accuracy	MCC
Majority voting	0.88	0.7	0.79	0.590
Stacking	0.87	0.83	0.85	0.701

#### 2.3.2. Comparison of Stacking-Based Ensemble Learning Method and the Individual RF Classifier

In order to verify the strength of the proposed ensemble method, the stacking-based ensemble method and the individual classifier for bacteriophage virion protein prediction are investigated. The prediction results are presented in Table 4. The stacking-based ensemble method returns a satisfactory performance with a little lower sensitivity, but higher specificity, accuracy and MCC, than those of the individual RF classifier.

**Table 4:** Performance of stacking-based ensemble learning method and the individual RF classifier.

Method	Sensitivity	Specificity	Accuracy	MCC
Individual RF	0.88	0.71	0.795	0.599
Stacking	0.87	0.83	0.85	0.701

**Figure 6:**
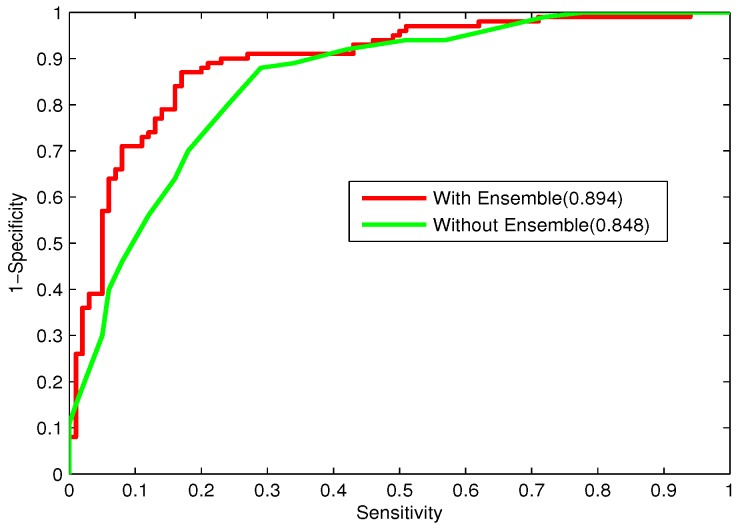
ROC curves with and without the stacking-based ensemble learning method.

Additionally, the ROC curves for the two models are depicted in Figure 6. From the figure, the curve for the stacking-based ensemble model is closer to the left side of the vertical axis, with a better AUC value of 0.894. Thus, the ensemble learning model gives a much better performance compared to the individual classifier.

### 2.4. Contribution of Feature Selection to the Stacking-Based Ensemble Method

**Figure 7:**
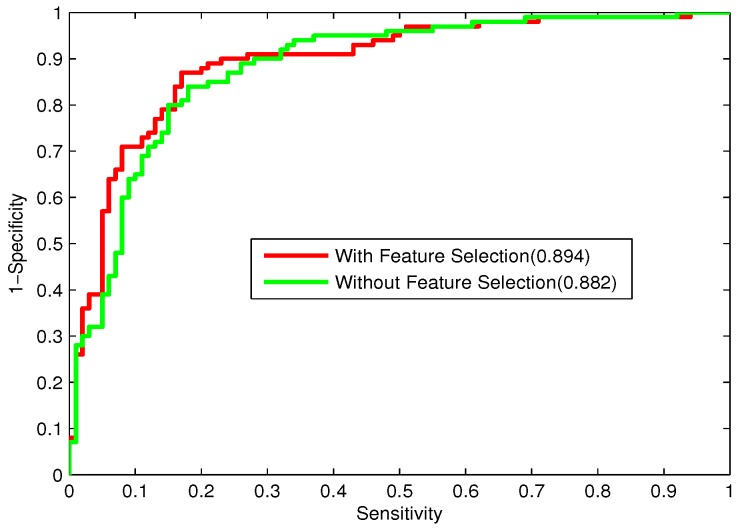
ROC curves of the stacking-based ensemble learning methods with and without feature selection.

To investigate the influence of feature selection on the performance of the stacking-based ensemble method, the prediction performance of the ensemble method with or without feature selection is shown in Table 5. Figure 7 depicts the ROC curves obtained with or without feature selection. As can be seen from Table 5 and Figure 7, the prediction results with feature selection outperform those without feature selection. There is a little improvement of the specificity and AUC obtained with feature selection, while the sensitivity, accuracy and MCC with feature selection are significantly better than those without feature selection. It appears that many redundant or uninformative features are present in the original feature sets, and Relief combined with IFS can significantly remove these useless features to greatly improve the performance of the ensemble model. The stacking-based ensemble learning method with feature selection is determined as the final predictor for bacteriophage virion protein prediction.

**Table 5:** Performance of stacking-based ensemble learning method with and without feature selection.

Method	Sensitivity	Specificity	Accuracy	MCC
Without feature selection	0.68	0.81	0.745	0.494
With feature selection	0.87	0.83	0.85	0.701

### 2.5. Comparisons with Existing Methods on the Independent Testing Dataset

To further assess the prediction performance of the proposed method, it is essential to compare the performance of the present model with the previous predictors on the independent testing dataset as given in Table S1. The performance comparison based on the same dataset is much more reliable, which can reflect the performance of a predictor more objectively. The performance comparison results are shown in Table 6. These results in Table 6 demonstrate that the sensitivity, accuracy and MCC obtained by the proposed method outperform those of [10,11]. In particular, the sensitivity, specificity, accuracy and MCC are 0.103, 0.195, 0.156 and 0.296 higher than those achieved by [10], respectively. Meanwhile, the outcomes of specificity yielded by our method and [11] are the same, but the sensitivity, accuracy and MCC of our method are significantly higher than those achieved by [11], which indicates that an unreasonable balance between sensitivity and specificity exists in [11]. In addition, it is important to highlight that the proposed method not only provides reasonable balance performance for positive and negative samples reflected by MCC, but also gives a satisfactory discrimination power expressed by accuracy. Overall, the proposed ensemble method is effective at predicting bacteriophage virion proteins, superior to previous methods.

**Table 6:** Comparison of prediction performance with existing methods on the independent testing dataset.

Reference	Sensitivity	Specificity	Accuracy	MCC
[10]	0.75	0.62	0.675	0.366
[11]	0.603	0.815	0.725	0.430
This study	0.853	0.815	0.831	0.662

The proposed method misclassifies 10 bacteriophage virion proteins on the independent testing dataset. Among the 10 bacteriophage virion proteins, six virion proteins are classified correctly by the previous method of [10] or the previous method of [11]. Four virion proteins are also misclassified by the previous methods [10,11], of which three virion proteins belong to part of the virion tail of prokaryotic viruses.

The proposed method misclassifies 17 bacteriophage non-virion proteins on the independent testing dataset. Among the 17 bacteriophage non-virion proteins, 12 non-virion proteins are classified correctly by the previous method [10] or previous method [11]. Five non-virion proteins are also misclassified by the previous methods [10,11], of which three non-virion proteins belong to host membrane proteins.

The above results indicate that it is difficult to predict bacteriophage protein sequences that are part of the virion tail or the host membrane. To further enhance the prediction performance, we will make an attempt to focus on the properties of theses proteins in future work. In addition, there are some bacteriophage virion proteins and non-virion proteins that are classified correctly by our method, but misclassified by the previous method of [10] or the previous method of [11]. Therefore, the proposed method can play a complementary role for distinguishing bacteriophage virion from non-virion proteins. Specific details about the prediction results of our method and previous methods on the independent testing dataset are available in Supplementary Table S2.

The promising performance of our predictor is mainly attributed to two aspects. One aspect is the rich informativeness of the feature vector, which includes discriminative sequence composition information, sequence order information, sequence statistical information and evolutionary information after the implemented feature selection procedure. The other aspect is the powerful stacking-based ensemble learning method.

### 2.6. Online Web Server

Since user-friendly and publicly-accessible web-servers represent the future direction for developing more useful predictors, a free web-server has been established at [19] to discriminate bacteriophage virion proteins from non-virion proteins. Users can enter query protein sequences in FASTA format or input the UniProtKB ID of the query protein sequences in the text box area for prediction. When protein sequences are submitted to the server, a job ID is presented to users. The predicted result page will return the input information and predicted result. The source code of our proposed method can be found in a supplementary file (Zip S1).

## 3. Materials and Methods

### 3.1. Data Collection

Bacteriophage virion protein sequences applied in this study are collected from the UniProt database [20] by searching “organism: phage AND subcellular location: virion AND reviewed: yes”. Due to the number of bacteriophage non-virion protein sequences being extremely large, protein sequences that were created after 2000 in the database and that match the search criterion “organism: phage NOT subcellular location: virion AND reviewed: yes” are selected as the bacteriophage non-virion proteins. Following the above procedures, 253 bacteriophage virion protein sequences and 248 bacteriophage non-virion protein sequences are obtained.

In order to obtain a reliable and high quality dataset, the following criteria are further performed. (1) The sequences that are fragments of other proteins are excluded because their information is redundant and lack integrity; (2) The sequences containing nonstandard letters, such as “B”, “X” or “Z”, are excluded, because their meanings are ambiguous.

It is well known that the benchmark dataset containing highly similar sequences can overestimate the performance and reduce the robustness of a predictor. Thus, a cutoff threshold is introduced to avoid homology bias and redundancy. The smaller the cutoff threshold is, the more stringent the benchmark dataset will be [21]. In order to avoid the smaller cutoff threshold value causing the benchmark dataset to be too small to have statistical significance, the sequence identity cutoff threshold is set as 80% to dislodge the redundant sequences using the CD-HIT program [22]. After the above screening procedures, the final benchmark dataset consists of 168 bacteriophage virion protein sequences and 192 bacteriophage non-virion protein sequences.

In order to validate the proposed ensemble model objectively, 100 bacteriophage virion proteins and 100 bacteriophage non-virion proteins are respectively selected from the final benchmark dataset as the training dataset for constructing the proposed model, while the rest of the 68 bacteriophage virion proteins and 92 bacteriophage non-virion proteins are the independent testing dataset. The training dataset and independent testing dataset are available in Supplementary Table S1.

### 3.2. Feature Extraction

To develop a powerful predictor for pattern recognition problems in bioinformatics, one of the key steps is to represent the protein sequences with appropriate descriptors that can truly reflect their intrinsic correlation with the target sequences to be predicted [21]. A single-feature descriptor cannot preserve enough discriminative information for protein attribute predictions, which may lead to unsatisfactory predictive performance. In such circumstances, the idea of hybrid features is adopted as a common way for enhancing the discrimination power of a predictor. In this study, hybrid features extracted from CTD, bi-profile Bayes, PseAAC and PSSM are utilized for encoding protein sequences.

#### 3.2.1. Composition, Transition and Distribution  

Amino acid composition is one of the most basic characteristics of proteins. To avoid the notorious dimensional disaster, the 20 native amino acids can be clustered into a smaller number of representative residues, called the reduced amino acid alphabet (RAAA) [23], as shown in Table 7.

Compared to the traditional amino acid composition, RAAA not only simplifies the complexity of the protein system, but also improves the ability to find structurally-conserved regions and the structural similarity of entire proteins [24].

The CTD method, introduced by Dubchak *et al*. [25], can effectively extract the global information of protein sequences. Recently, the CTD method has been widely applied to protein function predictions and structure studies [26,27]. Based on the cluster profiles (CP) in Table 7, three global descriptors, composition, transition and distribution, are employed in this study to describe the properties of protein sequences.

**Table 7:** Scheme for the reduced amino acid alphabets.

Reference	Cluster Profiles	Reduced Amino Acid Alphabets
[23]	CP(5)	G-IVFYW-ALMEQRK-P-NDHSTC
[28]	CP(6)	RDENQKH-LIVAMF-STYW-P-G-C
[29]	CP(7)	AILVGP-DE-HKR-FWY-NQ-ST-CM
[23]	CP(8)	G-IV-FYW-ALM-EQRK-P-ND-HSTC
[23]	CP(9)	G-IV-FYW-ALM-EQRK-P-ND-HS-TC
[30]	CP(10)	FWY-DE-H-K-R-C-M-QN-ST-AGILVP
[23]	CP(11)	G-IV-FYW-A-LM-EQRK-P-ND-HS-T-C

For a given $CP\left(n\right),n\in \left\{5,6,\cdots ,11\right\}$, the composition is defined as:

(1) $$\left(\frac{N_{1}}{L},\frac{N_{2}}{L},...,\frac{N_{n}}{L}\right)$$

where $N_{i},i\in \left\{1,2,\cdots ,n\right\}$ denotes the number of each group in CP(n) and *L* is the length of the whole sequence.

For a given $CP\left(n\right),n\in \left\{5,6,\cdots ,11\right\}$, the transition descriptor is defined as:

(2) $$T_{\alpha_{i}\alpha_{j}}=\frac{N_{\alpha_{i}\alpha_{j}}+N_{\alpha_{j}\alpha_{i}}}{L}$$

where i,j∈{1,2,⋯,n} and i≠j. $\alpha_{i}$ is one of the amino acid groups in CP(n). $N_{\alpha_{i}\alpha_{j}}$ is the number of the dipeptide encoded as “$\alpha_{i}\alpha_{j}$”.

Distribution measures the respective locations of the first, 25%, 50%, 75% and 100% of each amino acid group in CP(n), which can be calculated by:

(3) $$\left(\frac{N_{1,1}}{L},...,\frac{N_{1,5}}{L},\frac{N_{2,1}}{L},...,\frac{N_{2,5}}{L},...,\frac{N_{n,1}}{L},...,\frac{N_{n,5}}{L}\right)$$

where $N_{i,1}$ is the chain length within which the first of the amino acids of group *i* is located. $N_{i,2},N_{i,3},N_{i,4}.N_{i,5}$ measure the chain lengths within which the 25%, 50%, 75% and 100% of the amino acids of group *i* are located, respectively.

Based on the 7 different cluster profiles in Table 7, the prediction results obtained by the proposed CTD features are investigated and compared.

#### 3.2.2. Bi-Profile Bayes  

Statistical differences usually exist in the position-specific amino acid composition of protein sequences between positive and negative datasets. In this study, the bi-profile Bayes method is utilized to extract the statistical information about the position-specific amino acid composition between positive and negative datasets.

A previous study has shown that signal peptides play a vital role in the structures and functions of proteins [31]. Generally, important targeting signal peptides often occur at the N-terminal and retention signal peptides for protein sorting at the C-terminal of proteins [32]. In order to extract significant signal peptide information of a protein, the sequence segments at N-terminus and C-terminus are considered.

For the two-class classification problem, suppose $C_{0}$ and $C_{1}$ correspond to the negative class and the positive class, respectively; the amino acid composition at each position of the N-terminus and C-terminus with the length of *m* is calculated by the following formula.

(4) $$(N_{1}^{j},N_{2}^{j},\cdots ,N_{i}^{j},\cdots ,N_{m}^{j};C_{1}^{j},C_{2}^{j},\cdots ,C_{i}^{j},...,C_{m}^{j}),j=0,1$$

where j=0 represents the amino acid information extracted from the negative dataset; j=1 represents the amino acid information extracted from the positive dataset; $N_{i}^{j}$ are the posterior probabilities of 20 native amino acids at the *i*-th position of the N-terminus, represented by $N_{i}^{j}={\left(N_{i,1}^{j},N_{i,2}^{j},\cdots ,N_{i,20}^{j}\right)}^{T}$; and $C_{i}^{j}$ are the posterior probabilities of 20 native amino acids at the *i*-th position of the C-terminus, formulated as $C_{i}^{j}={\left(C_{i,1}^{j},C_{i,2}^{j},\cdots ,C_{i,20}^{j}\right)}^{T}$.

Based on the bi-profile Bayes method, a test protein sample can be formulated as:

(5) $$\left(P_{N}^{1},P_{N}^{2},\cdots ,P_{N}^{m};P_{C}^{1},P_{C}^{2},\cdots ,P_{C}^{m};P_{N}^{m+1},P_{N}^{m+2},\cdots ,P_{N}^{m+m};P_{C}^{m+1},P_{C}^{m+2},\cdots ,P_{C}^{m+m}\right)$$

where $\left(P_{N}^{1},P_{N}^{2},\cdots ,P_{N}^{m}\right)$ denote the posterior probabilities of each amino acid at each position of the N-terminus of the test protein sample in the positive dataset, which are extracted from $\left(N_{1}^{1},N_{2}^{1},\cdots ,N_{i}^{1},\cdots ,N_{m}^{1}\right)$; $(P_{C}^{1},P_{C}^{2},\cdots ,$ $P_{C}^{m})$ and denote the posterior probabilities of each amino acid at each position of the C-terminus of the test protein sample in the positive dataset, which are extracted from $\left(C_{1}^{1},C_{2}^{1},\cdots ,C_{i}^{1},\cdots ,C_{m}^{1}\right)$.

Similarly, $\left(P_{N}^{m+1},P_{N}^{m+2},\cdots ,P_{N}^{m+m}\right)$, extracted from $\left(N_{1}^{0},N_{2}^{0},\cdots ,N_{i}^{0},\cdots ,N_{m}^{0}\right)$, represent the posterior probabilities of each amino acid at each position of the N-terminus in the negative dataset; and $\left(P_{C}^{m+1},P_{C}^{m+2},\cdots ,P_{C}^{m+m}\right)$, extracted from $\left(C_{1}^{0},C_{2}^{0},\cdots ,C_{i}^{0},\cdots ,C_{m}^{0}\right)$, represent the posterior probabilities of each amino acid at each position of the C-terminus in the negative dataset.

The length of the N- or C-terminus can affect the predictive performance of a classifier. Therefore, different lengths of the N- or C-terminus are investigated variably from 15 to 25 residues to find out the discriminative one.

#### 3.2.3. Pseudo-Amino Acid Composition

Physicochemical properties of amino acids have a crucial influence on protein structures and functions [33]. Therefore, extracting features from protein sequences based on appropriate physicochemical properties may prospectively improve the performance of a predictor.

Previous studies have shown that certain physicochemical properties are related to the functions and structures of bacteriophage virion proteins. Coia *et al*. [34] have revealed that amino acids with small side-chain masses are prone to occur in bacteriophage virion proteins. Marvin *et al*. [35] have demonstrated that certain physicochemical properties, such as amphipathicity and charge, play vital roles in the functions of bacteriophage virion proteins. Kuzmicheva *et al*. [36] have reported that bacteriophage virion proteins should preserve small amino acids sharing diminutive positively-charged surfaces and higher occupied molecular orbitals.

Sequence order information represents important properties of protein sequences for prediction purposes, which may affect the predictive performance of a predictor [37]. The PseAAC approach, proposed by Chou [38], takes into consideration physicochemical properties and sequence order information of protein sequences.

In order to extract discriminative physicochemical features of protein sequences, 10 widely-used physicochemical properties of amino acids are adopted from the AAIndex (amino acid index) database [39] to calculate the correlations between residues along a protein sequence using the PseAAC method. They are the number of atoms, the number of potential hydrogen bonds, the number of electrostatic charges, the isoelectric point, mass, flexibility, hydrophilicity, hydrophobicity, propensity and the electron-ion interaction potential.

In terms of the 10 physicochemical properties mentioned above, based on PseAAC, a given protein sequence with *L* amino acids can be formulated as:

(6) $$P={\left(\psi_{1}^{1},\psi_{1}^{2},\cdots ,\psi_{1}^{\gamma },\psi_{2}^{1},\psi_{2}^{2},\cdots ,\psi_{2}^{\gamma },\cdots ,\psi_{10}^{1},\psi_{10}^{2},\cdots ,\psi_{10}^{\gamma }\right)}^{T}$$

(7) $$\psi_{j}^{\gamma }=\frac{1}{L-\gamma }\sum_{i=1}^{L-\gamma }p_{i}^{j}p_{i+\gamma }^{j},\left(j=1,2,...,10,0<\gamma <L\right)$$

where *T* denotes a transpose operator; $\psi_{j}^{\gamma }$ is the γ-th tier correlation factor based on the *j*-th physicochemical property; and $p_{i}^{j}$ is the *j*-th physicochemical property value of the *i*-th residue along the protein sequence. γ is closely related to sequence order information and plays an important role in the performance of a predictor. Therefore, we evaluate the discrimination power of the features based on different γ varying from 1 to 10.

#### 3.2.4. Position-Specific Scoring Matrix  

Protein sequences have stemmed from a very finite number of ancestral species protein sequences, involving changes, insertions and deletions of single or several residues [40]. As a result, the original and resultant protein sequences may have few sequence similarities, but they may still share some structure similarities and the same functions [41]. Therefore, evolutionary conservations can determine important biological functions and contribute to biological analysis [42].

The position-specific scoring matrix (PSSM) is adopted in this study to extract evolution information, which has been widely employed in protein attribute prediction problems [43,44]. To obtain the PSSM profiles, the Position-Specific Iterative BLAST (PSI-BLAST) [45], a powerful sequence searching program, is applied to search the non-redundant (NR) database for multiple sequence alignment through 3 iterations using the E-value cutoff of 0.0001. For a given protein sequence with *L* amino acids, the corresponding PSSM has L×20 elements and is defined as:

(8) $$P_{\mathrm{PSSM}}=\left[\begin{array}{cccccc} E_{1\to 1} & E_{1\to 2} & \cdots & E_{1\to j} & \cdots & E_{1\to 20}\\ E_{2\to 1} & E_{2\to 2} & \cdots & E_{2\to j} & \cdots & E_{2\to 20}\\ \vdots & \vdots & \cdots & \vdots & \cdots & \vdots \\ E_{i\to 1} & E_{i\to 2} & \cdots & E_{i\to j} & \cdots & E_{i\to 20}\\ \vdots & \vdots & \cdots & \vdots & \cdots & \vdots \\ E_{L\to 1} & E_{L\to 2} & \cdots & E_{L\to j} & \cdots & E_{L\to 20} \end{array}\right]$$

where the values in the *i*-th row are the probabilities of the *i*-th residue in a given protein sequence mutating to 20 native amino acids. Then, the PSSM is normalized with the following sigmoid function to scale each element to a range of 0 to 1:

(9) $$f\left(x\right)=\frac{1}{1+e^{-x}}$$

where *x* is the original PSSM value.

To obtain a uniform number of features, we sum up all of the columns and rows in the PSSM corresponding to the amino acids in the same group of cluster profiles as indicated in Section 3.2.1. For the cluster profile with *N* groups, the PSSM profile is transformed to a N×N matrix following the above procedures. The elements of the N×N matrix are employed to encode each protein sequence. The discrimination power of the proposed PSSM conservation scores based on 7 cluster profiles is evaluated, respectively. Then, the cluster profile that achieves the best performance is chosen to extract features from PSSM.

### 3.3. Feature Selection

Through the feature extraction methods, protein sequences are encoded into various numerical feature vectors. However, not all of the extracted features contribute to the prediction. Much information redundancy or noise may exist in the extracted features, which will deteriorate the performance of a predictor and result in dimensional disaster [46].

Feature selection is essential to overcome the above troubles. It is a critical step in the classification problems to select the discriminative features and decrease model complexity, which can not only lower computation complexity, but also build a high-quality and robust prediction model [47]. In this study, the Relief algorithm combined with IFS is employed to acquire the optimal feature subsets of individual feature spaces.

Relief: The Relief algorithm, originally proposed by Kira [48], is used to depict the relevance between the features and class labels. Based on the ability of the feature to distinguish the near samples, the Relief algorithm is a feature-weighting algorithm that can be used to estimate the quality of each feature. During the process, the Relief algorithm endows each feature with a weighting iteratively as formulated by:

(10) $$W_{p}^{i+1}=W_{p}^{i}-\frac{diff(Y,x_{i},H\left(x_{i}\right))}{m}+\frac{diff(S,x_{i},M\left(x_{i}\right))}{m}$$

(11) $$diff\left(*,x,y\right)=\left\{\begin{array}{c} ∥x-y∥\;\;\;,\;x\neq y\\ \;\;\;\;\;\;\;\;\;\;\;0,\;\;\;\;\;\;\;\;\;\;\;\;\;\;\;\;\;\;\;\;\;\;\;\;\;\;\;\;\;\;x=y \end{array}\right.$$

where $W_{p}^{i}$ and $W_{p}^{i+1}$ are separately the current and next weighting values. *p* stands for a given feature, and $x_{i}$ denotes the *i*-th sample sequence. $H(x_{i})$, referred to as a near hit, represents the nearest neighbor samples from the same class label against $x_{i}$. $M(x_{i})$, referred to as a near miss, represents the nearest neighbor samples from the different class labels against $x_{i}$. *Y* and *S* are the sample sets with the same and different class labels against $x_{i}$, respectively. *m* is the number of random samples, and the function of diff(*,x,y) is used for calculating the distance between the random samples.

The ranked feature list can be obtained based on $W_{p},p=1,2,...,N$, represented as:

(12) $$F=\{f_{1},f_{2},...,f_{N}\}$$

where $f_{1}$ represents the feature with the highest value of $W_{p}$, $f_{2}$ with the second highest value of $W_{p}$ ,⋯, and $f_{N}$ with the lowest value of $W_{p}$.

Incremental feature selection: Based on the ranked feature list according to their relevance to the classes evaluated by Relief, IFS, one of the well-known searching strategies of feature selection, is employed to determine the optimal feature subset.

The IFS procedure starts with an empty subset and adds features in the ranked feature list one by one from higher to lower rank into the feature subset [49]. A new subset is generated when a new feature in the ranked list is added, and the *i*-th feature subset can be formulated as:

(13) $$F_{i}=\left\{f_{1},f_{2},\cdots ,f_{i}\right\}\left(1\leq i\leq N\right)$$

For each feature subset $F_{i}$, an RF-based predictor is constructed and evaluated by 10-fold cross-validation. The IFS curve can be drawn with accuracy values as the *y*-axis and index *i* of $F_{i}$ as the *x*-axis. The optimal feature subset is obtained when the IFS curve reaches its peak.

### 3.4. Machine Learning Method

Random Forest: The RF method, developed by L. Breiman [50], is a popular machine learning method that has been successfully employed in various biological prediction problems [51,52]. It is an ensemble classifier generating multiple decision trees, where each decision tree is constructed based on the training dataset and produces a classification label. To predict a test sample, its feature vector is put into each of the decision trees in the forest, and each tree gives a vote suggesting one class. Then, the RF method will choose the final classification label with the most votes over all of the decision trees as the output class [53]. Refer to [50] for a detailed description of the RF method. In this study, 4 random forest models are developed, and each one is trained by the individual feature space described in Section 3.2.

Logistic regression: Logistic regression (LR), a probabilistic statistical classification model, is a machine learning framework suitable to establish the logistic relationship between the two class labels and the numerical feature vectors [54]. It has been widely applied to various pattern recognition problems, such as the prediction of flexible regions in proteins [55], the determination of protein folding kinetic types [56] and the prediction of lysine acetylation site [33].

Given a protein sequence and its feature vector, the likelihood can be calculated by the logistic regression model. The feature vector $F=(f_{1},f_{2},...,f_{N})$ combined with the class label is used as the input:

(14) $$P=\frac{1}{1+e^{-(\theta_{0}+\theta_{1}f_{1}+\theta_{2}f_{2}+...+\theta_{N}f_{N})}}$$

where the optimized regression coefficients $\theta_{0},\theta_{1},...\theta_{N}$ are obtained based on the training dataset.

In this study, the logistic regression approach integrates the outputs of the 4 RF models to provide the final prediction score *P*, which is formulated as:

(15) $$P=\frac{1}{1+e^{-(\theta_{0}+\theta_{1}S_{1}+\theta_{2}S_{2}+\theta_{3}S_{3}+\theta_{4}S_{4})}}$$

where the constant term $\theta_{0}$ and the coefficient $\theta_{i}$ of each RF output $S_{i}$ are deduced through the regression process based on the training dataset.

For protein sequences in the test dataset, *P* has values between 0 and 1, which will be optimized to maximize the accuracy for bacteriophage virion protein prediction.

The WEKA (Waikato Environment for Knowledge Analysis) software package [57] is used for the above-mentioned machine learning algorithms, where default parameters are employed for 4 RF models.

### 3.5. Ensemble Learning Method

Every single classifier has its own shortcomings, and it will not always perform well on all datasets [14]. Ensemble learning emerges as a promising measure to overcome this problem. Unlike the single-prediction algorithm, ensemble learning consists of multiple basic individual classifiers and then aggregates the outputs of all independent classifiers to tackle the same classification task [58]. Due to the procedure of consulting several experts for suggestions before giving the final results, the ensemble learning is supposed to significantly improve the performance of a prediction method [59].

In this study, 4 different random forest models as the first layer are constructed. Then, two popular methods, majority voting and stacking, are employed to combine the 4 random forest models to give the final prediction results.

Majority voting: The majority voting strategy, the simplest way to combine the predicted class labels of multiple classifiers [60], is used to aggregate the independent classifiers. The target protein sequence is assigned to the predicted class label whose score function has the maximum value, that is the corresponding class label achieves the highest number of votes [60].

For a query protein, denote the output of the *i*-th RF model by:

(16) $$s_{i,j}\in \left\{-1,1\right\},i=1,2,...,4;j=1,2$$

where *j* denotes the predicted class label. $s_{i,1}=1,s_{i,2}=-1$ separately represent the sample predicted as the positive class $C_{1}$ and the sample predicted as the negative class $C_{2}$.

The output of the 4 RF models is given as a 4-dimensional vector ${\left[s_{1,j},s_{2,j},s_{3,j},s_{4,j}\right]}^{T}$. The query protein is assigned to the positive class when S≥0 according to the following formula:

(17) $$S=\sum_{i=1}^{4}s_{i,j}$$

Otherwise, it is predicted as the negative class.

Stacking: Stacking is an approach for combining multiple classifiers and achieves the highest generalization accuracy [61]. Compared to the majority voting method, stacking will learn twice in two layers. In the first layer, a number of individual classifiers are generated using the original feature vectors as the input. In the second layer, a specific classifier is employed to combine the predicted probabilities for every class label from the classifiers in the first layer [62].

In this study, 4 random forest models are generated as the first layer, each of which is trained by 4 different feature spaces, respectively. Then, the logistic regression approach integrates the 4 independent prediction scores from the first layer to give the final results.

Majority voting and stacking both have their own particular merits. The former is the simplest combination strategy, without cross-validation and the training of the second-layer classifier, which is computationally inexpensive. The latter provides the function of being highly extensible to more classifier algorithms at both the first and second layers, regardless of their internal structures [62].

In the study, the performance of majority voting and stacking will be compared.

### 3.6. Performance Measures

The independent dataset test, the sub-sampling test (e.g., 5-fold or 10-fold cross-validation) and the jackknife test are widely applied to examine the performance of a predictor [63]. The jackknife test is deemed as the most objective method in statistical prediction, which can always obtain a unique outcome for a given benchmark dataset [59]. To reduce the computational complexity, 10-fold cross-validation is adopted in this study. During 10-fold cross-validation, the training dataset is randomly divided into 10 equally-sized folds. Each fold is used as the testing set, and the remaining 9 folds as the training set. This process is repeated 10 times with different combinations of training and testing datasets. Then, the ultimate result is obtained by averaging the 10 prediction results.

To illuminate the performance of the predictor more intuitively, 4 common measurements: sensitivity (Sn), specificity (Sp), accuracy (Acc) and Matthew’s correlation coefficient MCC are adopted, which are formulated as:

(18) $$S_{n}=\frac{TP}{TP+FN}$$

(19) $$S_{p}=\frac{TN}{TN+FP}$$

(20) $$Acc=\frac{TP+TN}{TP+FP+TN+FN}$$

(21) $$MCC=\frac{TP*TN-FP*FN}{\sqrt{\left(TP+FN)(TP+FP)(TN+FP)(TN+FN\right)}}$$

where TP, TN, FP and FN stand for the numbers of true positive, true negative, false positive and false negative, respectively.

To further evaluate the performance of the classifier, the receiver operating characteristic (ROC) curve, one of the most valid measures in pattern recognition, is also employed [64]. The ROC curve is plotted with the Sn as the *y*-axis and 1-Sp as the *x*-axis by varying the thresholds. The area under the ROC curve (AUC) is the index for model evaluation obtained from the ROC curve. The higher AUC value corresponds to the better performance of the classifier.

## 4. Conclusions

Bacteriophage virion proteins have the functions of specificity determination for the host bacteria, antigenicity of bacteriophages and clinical phage therapy. Due to the major functions of bacteriophage virion proteins in deciding applications of bacteriophages, identifying bacteriophage virion proteins is crucial for understanding the relationship between bacteriophage and host bacteria and the influence of bacteriophages on the development of new pathogens and antibacterial drugs. It is urgent to develop computational methods for rapidly and effectively identifying bacteriophages virion proteins. An ensemble method for bacteriophage virion protein prediction has been presented in this study with hybrid features extracted from CTD, bi-profile Bayes, PseAAC and PSSM. To solve the dimensional disaster and to improve the performance, Relief combined with the IFS method is adopted to obtain the optimal feature subsets. The AUCs of individual RF modules based on four individual optimal features are 0.845, 0.888, 0.823 and 0.822, respectively, indicating the discrimination power of the selected features. The four RF models are combined by a stacking strategy with an accuracy of 0.85. To evaluate the prediction performance objectively, the proposed ensemble method is tested on the independent testing dataset and compared to previous studies. The prediction results of our proposed method are superior to those of the earlier studies. Thus, the proposed method may provide a useful tool for understanding the relationship between bacteriophage and host bacteria. Furthermore, it may provide crucial clues to develop new pathogens and antibacterial drugs in the future.

## Supplementary Materials

Supplementary materials can be found at http://www.mdpi.com/1422-0067/16/09/21734/s1.

## References

[1] Seguritan V., Alves N., Arnoult M., Raymond A., Lorimer D., Burgin A.B., Salamon P., Segall A.M. (2012). Artificial neural networks trained to detect viral and phage structural proteins. PLoS Comput. Biol..

[2] Denton C., Crosby R.J. (2013). Bacteriophages: Biology, Applications and Role in Health and Disease.

[3] Schaechter M. (2009). Desk Encyclopedia of Microbiology.

[4] Hanlon G.W. (2007). Bacteriophages: An appraisal of their role in the treatment of bacterial infections. Int. J. Antimicrob. Agents.

[5] Martelet A., L’Hostis G., Tavares P., Brasiles S., Fenaille F., Rozand C., Theretz A., Gervasi G., Tabet J.C., Ezan E. (2014). Bacterial detection using unlabeled phage amplification and mass spectrometry through structural and nonstructural phage markers. J. Proteome Res..

[6] Aguilar P.V., Adams A.P., Wang E., Kang W., Carrara A.S., Anishchenko M., Frolov I., Weaver S.C. (2008). Structural and nonstructural protein genome regions of eastern equine encephalitis virus are determinants of interferon sensitivity and murine virulence. J. Virol..

[7] Moreland N.J., Tay M.Y.F., Lim E., Paradkar P.N., Doan D.N.P., Yau Y.H., Shochat S.G., Vasudevan S.G. (2010). High affinity human antibody fragments to dengue virus non-structural protein 3. PLoS Negl. Trop. Dis..

[8] Clokie M.R., Thalassinos K., Boulanger P., Slade S.E., McPhie S.S., Cane M., Scrivens J.H., Mann N.H. (2008). A proteomic approach to the identification of the major virion structural proteins of the marine cyanomyovirus S-PM2. Microbiology.

[9] Li J., Saman K.H., Kells C.I., Tang S.L. (2007). Gene function prediction based on genomic context clustering and discriminative learning: An application to bacteriophages. BMC Bioinform..

[10] Feng P.M., Ding H., Chen W., Lin H. (2013). Naïve bayes classifier with feature selection to identify phage virion proteins. Comput. Math. Methods Med..

[11] Ding H., Feng P.M., Chen W., Lin H. (2014). Identification of bacteriophage virion proteins by the ANOVA feature selection and analysis. Mol. Biosyst..

[12] Zhang Y.N., Yu D.J., Li S.S., Fan Y.X., Huang Y., Shen H.B. (2012). Predicting protein-ATP binding sites from primary sequence through fusing bi-profile sampling of multi-view features. BMC Bioinform..

[13] Han G.S., Yu Z.G., Anh V., Krishnajith A.P.D., Tian Y.C. (2013). An ensemble method for predicting subnuclear localizations from primary protein structures. PLoS ONE.

[14] Zou C.X., Gong J.Y., Li H.L. (2013). An improved sequence based prediction protocol for DNA-binding proteins using SVM and comprehensive feature analysis. BMC Bioinform..

[15] Yang R.T., Zhang C.J., Gao R., Zhang L.N. (2015). An ensemble method with hybrid features to identify extracellular matrix proteins. PLoS ONE.

[16] Li L.Q., Zhang Y., Zou L.Y., Li C.Q., Yu B., Zheng X.Q., Zhou Y. (2012). An ensemble classifier for eukaryotic protein subcellular location prediction using gene ontology categories and amino acid hydrophobicity. PLoS ONE.

[17] Xie H.L., Fu L., Nie X.D. (2013). Using ensemble SVM to identify human GPCRs *N*-linked glycosylation sites based on the general form of Chou’s PseAAC. Protein Eng. Des. Sel..

[18] Rokach L. (2010). Ensemble-based classifiers. Artif. Intell. Rev..

[19] An Ensemble Method for Predicting Bacteriophage Virion Proteins. http://pbvp.weka.cc.

[20] Magrane M., UniProt Consortium (2011). UniProt Knowledgebase: A hub of integrated protein data. Database (Oxf.).

[21] Chou K.C. (2011). Some remarks on protein attribute prediction and pseudo amino acid composition. J. Theor. Biol..

[22] Li W., Jaroszewski L., Godzik A. (2001). CD-HIT: A fast program for clustering and comparing large sets of protein or nucleotide sequences. Bioinformatics.

[23] Solis A.D., Rackovsky S. (2000). Optimized representations and maximal information in proteins. Protein.

[24] Feng P.M., Chen W., Lin H., Chou K.C. (2013). iHSP-PseRAAAC: Identifying the heat shock protein families using pseudo reduced amino acid alphabet aomposition. Anal. Biochem..

[25] Dubchak I., Muchnik I., Holbrook S.R., Kim S.H. (1995). Prediction of protein folding class using global description of amino acid sequence. Proc. Natl. Acad. Sci. USA.

[26] Cai C.Z., Han L.Y., Ji Z.L., Chen X., Chen Y.Z. (2003). SVM-Prot: Web-based support vector machine software for functional classification of a protein from its primary sequence. Nucleic Acids Res..

[27] Cui J., Han L.Y., Lin H.H., Zhang H.L., Tang Z.Q., Zheng C.J., Cao Z.W., Chen Y.Z. (2007). Prediction of MHC-binding peptides of flexible lengths from sequence-derived structural and physicochemical attributes. Mol. Immunol..

[28] Chen W., Feng P., Lin H. (2012). Prediction of ketoacyl synthase family using reduced amino acid alphabets. J. Ind. Microbiol. Biotechnol..

[29] Fan G.L., Li Q.Z. (2012). Predicting protein submitochondria locations by combining different descriptors into the general form of Chou’s pseudo amino acid composition. Amino Acids.

[30] Sun C., Shi Z.Z., Zhou X.B., Chen L.N., Zhao X.M. (2013). Prediction of *S*-glutathionylation sites based on protein sequences. PLoS ONE.

[31] Kaundal R., Raghava G.P. (2009). RSLpred: An integrative system for predicting subcellular localization of rice proteins combining compositional and evolutionary information. Proteomics.

[32] Emanuelsson O., Nielsen H., Brunak S., Heijne G.V. (2000). Predicting subcellular localization of proteins based on their N-terminal amino acid sequence. J. Mol. Biol..

[33] Hou T., Zheng G.Y., Zhang P.Y., Jia J., Li J., Xie L., Wei C.C., Li Y.X. (2014). LAceP: Lysine acetylation site prediction using logistic regression classifiers. PLoS ONE.

[34] Coia G., Parker M.D., Speight G., Byrne M.E., Westaway E.G. (1988). Nucleotide and complete amino acid sequences of Kunjin virus: Definitive gene order and characteristics of the virus-specified proteins. J. Gen. Virol..

[35] Marvin D.A., Hale R.D., Nave C., Citterich M.H. (1994). Molecular models and structural comparisons of native and mutant class I filamentous bacteriophages Ff (fd, f1, M13), If1 and IKe. J. Mol. Biol..

[36] Kuzmicheva G.A., Jayanna P.K., Eroshkin A.M., Grishina M.A., Pereyaslavskaya E.S., Potemkin V.A., Petrenko V.A. (2009). Mutations in fd phage major coat protein modulate affinity of the displayed peptide. Protein Eng. Des. Sel..

[37] Garg A., Gupta D. (2008). VirulentPred: A SVM based prediction method for virulent proteins in bacterial pathogens. BMC Bioinform..

[38] Chou K.C. (2001). Prediction of protein cellular attributes using pseudo amino acid composition. Proteins Struct. Funct. Bioinform..

[39] Kawashima S., Kanehisa M. (2000). AAindex: Amino acid index database. Nucleic Acids Res..

[40] Chou K.C. (2004). Review: Structural bioinformatics and its impact to biomedical science. Curr. Med. Chem..

[41] Li B.Q., Hu L.L., Chen L., Feng K.Y., Cai Y.D., Chou K.C. (2012). Prediction of protein domain with mRMR feature selection and analysis. PLoS ONE.

[42] Niu S., Hu L.L., Zheng L.L., Huang T., Feng K.Y., Cai Y.D., Li H.P., Li Y.X., Chou K.C. (2012). Predicting protein oxidation sites with feature selection and analysis approach. J. Biomol. Struct. Dyn..

[43] Kumar M., Gromiha M., Raghava G. (2007). Identification of DNA-binding proteins using support vector machines and evolutionary profiles. BMC Bioinform..

[44] Chen S.A., Ou Y.Y., Lee T.Y., Gromiha M.M. (2011). Prediction of transporter targets using efficient RBF networks with PSSM profiles and biochemical properties. Bioinformatics.

[45] Altschul S.F., Madden T.L., Schaffer A.A., Zhang J.H., Zhang Z., Miller W., Lipman D.J. (1997). Gapped BLAST and PSI-BLAST: A new generation of protein database search programs. Nucleic Acids Res..

[46] Qian J., Miao D.Q., Zhang Z.H., Li W. (2011). Hybrid approaches to attribute reduction based on indiscernibility and discernibility relation. Int. J. Approx. Reason..

[47] Wang P., Xiao X. (2014). NRPred-FS: A feature selection based two level predictor for nuclear receptors. J. Proteom. Bioinform..

[48] Kira K., Rendell L.A. The feature selection problem: Traditional methods and a new algorithm. Proceedings of the Tenth National Conference on Artificial Intelligence.

[49] Lin H., Chen W., Ding H. (2013). AcalPred: A sequence-based tool for discriminating between acidic and alkaline enzymes. PLoS ONE.

[50] Breiman L. (2001). Random forests. Mach. Learn..

[51] Jia S.C., Hu X.Z. (2011). Using random forest algorithm to predict β-hairpin motifs. Protein Pept. Lett..

[52] Zhang N., Li B.Q., Gao S., Ruan J.S., Cai Y.D. (2012). Computational prediction and analysis of protein γ-carboxylation sites based on a random forest method. Mol. Biol. Syst..

[53] Lou W.C., Wang X.Q., Chen F., Chen Y.X., Jiang B., Zhang H. (2014). Sequence based prediction of dna-binding proteins based on hybrid feature selection using random forest and gaussian Naïve Bayes. PLoS ONE.

[54] Bishop C.M. (2006). Pattern Recognition and Machine Learning.

[55] Chen K., Kurgan L.A., Ruan J. (2007). Prediction of flexible/rigid regions in proteins from sequences using collocated amino acid pairs. BMC Struct. Biol..

[56] Huang J.T., Cheng J.P. (2008). Differentiation between two-state and multi-state folding proteins based on sequence. Proteins.

[57] Witten I.H., Frank E. (2005). Data mining: Practical machine learning tools and techniques.

[58] Xu R.F., Zhou J.Y., Liu B., Yao L., He Y.H., Zou Q., Wang X.L. (2014). enDNA-Prot: Identification of dna-binding proteins by applying ensemble learning. BioMed Res. Int..

[59] Chou K.C., Shen H.B. (2007). Recent progress in protein subcellular location prediction. Anal. Biochem..

[60] Rutaa D., Gabrysb B. (2005). Classifier selection for majority voting. Inf. Fusion.

[61] Xia J.F., Zhao X.M., Huang D.S. (2010). Predicting protein–protein interactions from protein sequences using meta predictor. Amino Acids.

[62] Chou K.C., Zhang C.T. (1995). Review: Prediction of protein structural classes. Crit. Rev. Biochem. Mol. Biol..

[63] Rutaa D., Gabrysb B. (2005). Classifier selection for majority voting. Inf. Fusion.

[64] Gribskov M., Robinson N.L. (1996). Use of receiver operating characteristic (ROC) analysis to evaluate sequence matching. J. Comput. Chem..

